# Internal jugular vein thrombosis presenting as a painful neck mass due to a spontaneous dislocated subclavian port catheter as long-term complication: a case report

**DOI:** 10.4076/1757-1626-2-7991

**Published:** 2009-06-09

**Authors:** Marcel Binnebösel, Jochen Grommes, Karsten Junge, Sonja Göbner, Volker Schumpelick, Son Truong

**Affiliations:** Department of Surgery, RWTH Aachen University HospitalGermany

## Abstract

Central venous access devices are extensively used for long-term chemotherapy and parenteral nutrition. However, there are some possible immediate, early, and late complications related to the implantation technique, care, and maintenance. We present the uncommon occurrence of a thrombosis of the internal jugular vein due to a spontaneous migration of a Port-A-Cath catheter into the ipsilateral internal jugular vein as a delayed complication of a central venous access catheter implanted for chemotherapy delivery. A review of the literature is given, and the factors responsible for this unusual complication will be discussed.

## Introduction

Central venous access devices are extensively used for long-term chemotherapy and parenteral nutrition. They have several advantages compared to other methods of venous access: they are easy to implant under local anaesthesia, have less discomfort for the patients, allow low costs, can be implanted in day cases, and can be managed ambulatory. However, there are some possible immediate, early, and late complications related to the implantation technique, care, and maintenance. Spontaneous migration as a late complication of central venous catheters after satisfactory initial placement is uncommon but is associated with a number of complications, including neck pain, shoulder pain, ear pain, infection, venous thrombosis, and neurological complications. We present the uncommon occurrence of a thrombosis of the internal jugular vein due to a spontaneous migration of a Port-A-Cath catheter into the ipsilateral internal jugular vein as a delayed complication of a central venous access catheter implanted for chemotherapy delivery. A review of the literature is given, and the factors responsible for this unusual complication will be discussed.

## Case presentation

**Figure 1.**
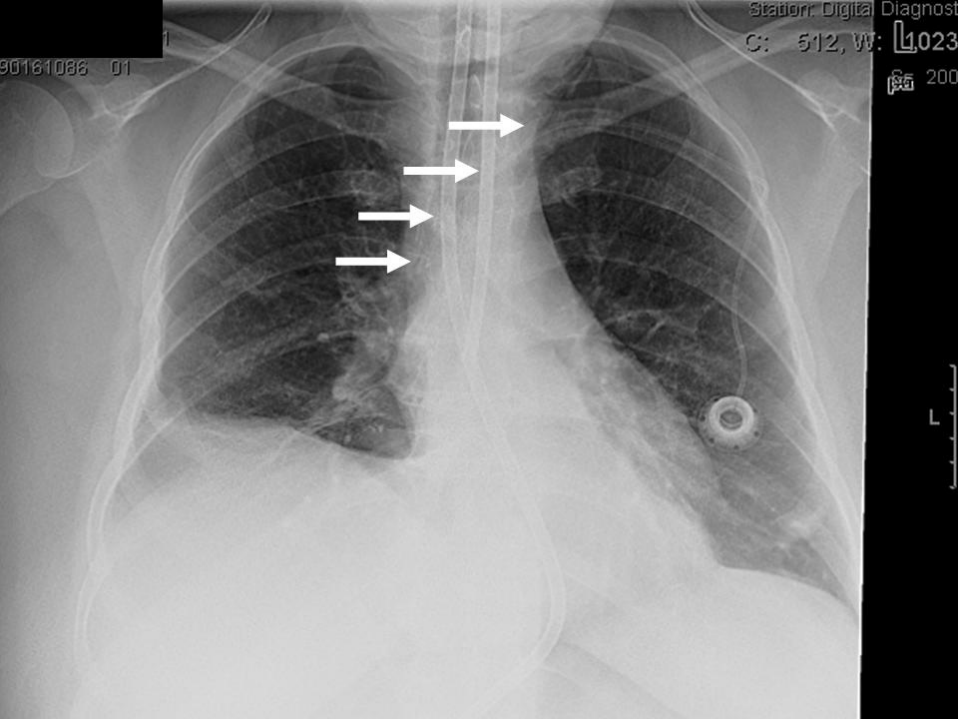
Radiograph of the chest following implantation of the Port-A-Cath catheter. The tip of the catheter was advanced to the distal part of the superior vena cava from the left subclavian vein (white arrows).

**Figure 2.**
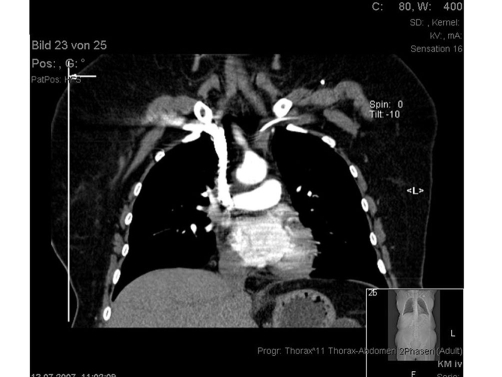
Staging CT-scan preoperatively demonstrating an accurate positioning of the catheter.

In a 44-year-old female Caucasian patient, adenocarcinoma of the oesophagus located in the gastro-oesophageal junction (33 to 39 cm) was diagnosed in August 2007. The disease was Stage III (T3 N1) because of adventitial involvement and proof of suspectly enhanced mediastinal and paraoesophageal lymph nodes. The patient underwent preoperative (neoadjuvant) chemotherapy using ECF (intravenous epirubicin 50 mg/m^2^ and cisplatin 60 mg/m^2^ every 3 weeks at day one respectively, with continuous infusion of 5-FU 200 mg/m^2^ per day). Three cycles of the intravenous chemotherapy were administered after implantation of a central venous access (Figures 1,2). The tip of the catheter was advanced to the distal part of the superior vena cava (SVC) from the left subclavian vein that was approached by percutaneous implantation using Seldinger technique in local anaesthesia. Following subcutaneous tunnelling the infusion port was embedded in front of the left pectoral muscle fascia.

Three month following the beginning of the neoadjuvant chemotherapy the patient proceeded to surgery. A proximal gastric resection and transhiatal subtotal oesophagectomy without thoracotomy through median laparotomy and left sided cervical incision was performed. Proximal gastric resection, oesophageal resection and reconstruction were performed in a single operation. The oesophageal substitute was positioned in the posterior mediastinum in the original oesophageal bed (gastric pull-up). Following the gastric pull-up a feeding nasogastric tube was positioned in the substitute and bilateral chest drains were performed. The patient was monitored up to the third postoperative day on the intensive care unit. All drains were gradually removed until the seventh postoperative day. The postoperative course was uneventful, except for a cervical wound healing complication due to a slight leakage of the gastro-oesophageal anastomosis. Temporarily feeding was conducted by nasogastric tube, and the leakage was successively occluded by fibrin sealant via endoscopy. The patient was hospitalised up to the 28^th^ postoperative day. One month following the operation endoscopy confirmed a healed cervical anastomosis. Therefore, the postoperative chemotherapy using ECF was resumed two month after the operation. Because of persistent vomiting and progressive loss of weight the intravenous chemotherapy was terminated after 2 cycles. Three and six months after termination of the oncological treatment, radiologically no recurrence was observed. Endoscopy did also not find any residual disease, anastomotic stenosis or signs of wound healing complications.

**Figure 3.**
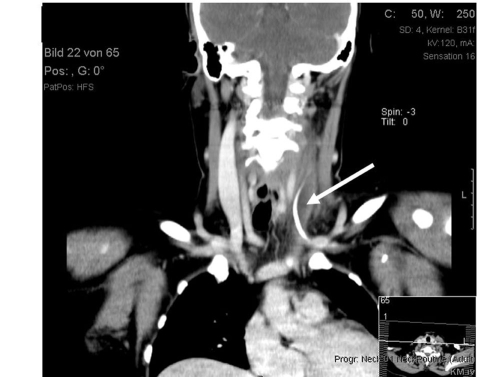
CT-scan representing thrombosis of the left internal jugular vein due to a dislocated left subclavian catheter (white arrows).

Eleven month after termination of the oncological treatment the patient was admitted to our outpatient clinic because of a left sided painful neck mass. The C-reactive protein was elevated up to 155 mg/l (reference range < 5), all other laboratory markers revealed no pathological results. An ultrasonic testing was performed and revealed a thrombosis of the left internal jugular vein due to a bar-shaped intravascular foreign body. Therefore, a computer tomography of the neck and upper thorax was arranged (Figure 3). CT scan confirmed thrombosis and demonstrated an extension of the thrombus up to the left subclavian vein, which showed proper perfusion. The bar-shaped intravascular foreign body could be revealed as ipsilateral dislocated Port-A-Cath catheter in continuity, whereas a catheter rupture could be excepted. Following preparation of the patient the Port-a-Cath catheter was removed surgically. Preoperatively a bolus of 5000 I.U. of an unfractionated heparin was administered intravenously; subsequently 25.000 I.U. of an unfractionated heparin per day were administered continuously intravenously. From the first postoperative day up to the third postoperative month the anticoagulation was performed orally (Phenprocoumon). The hospitalisation was terminated at the third postoperative day.

## Discussion

Utilizing Porth-A-Cath catheters in clinical practice immediate or early complications as well as delayed complications are known. Immediate or early complications are frequently related to clinical conditions of the neoplastic patients, such as necrosis of the skin at the port side, infection of subcutaneous pocket, and infections of the port system. In the reviewed literature vascular catheter infections and catheter thrombosis are the two most common and serious complications, reported in 0-7.7 and 1.5-13%, respectively [1]. Central venous catheter primary displacement as early complication is not an uncommon event and can occur during catheter insertion. The catheter tip, usually advanced as far as the vena cava-right atrial junction, can shift to another venous district. The incidence of such a displacement depends on the approach. In case of a subclavicular approach, like it was performed in our patient, the rate of catheter primary displacement is stated 30% for the external jugular vein, and 5.7% for the internal jugular vein [2,3]. In our case an immediate or early dislocation can be excepted, because both following implantation of the catheter and in the CT-scan before the operation the correct position of the catheter tip could be confirmed.

Furthermore, delayed complications of Port-A-Cath catheter are known. Catheter disconnection and embolization is a rare long-term complication reported in 0.9-1.7% [4]. Spontaneous intravascular fracture of the outlet catheter is reported in the literature in 0.6-8% of the cases [4]. The catheter rupture is often due to the wear over the costoclavicular angle. Beside an immediate migration of the catheter from its initial postoperative position, it can occur several days or month after insertion. In the reviewed literature the incidence is reported in 2.5-2.6% of the [1,5].

The proper spontaneous migration mechanism is still unknown. In the reviewed literature several hypotheses are given, such as forced flushing, upper extremity vigorous movements, neck flexion, congestive heart failure, changing in thoracic pressure with coughing and vomiting [1,6-8]. Quick thoracic pressure increasing during coughing or vomiting could cause a temporary venous flux inversion and determine displacement of the catheter tip. In our patient a persistent episode of vomiting was existent, and as such the main cause of premature abruption of ECF intravenous chemotherapy. We speculate that the migration in our patient was related to severe vomiting, and thus vigorous changes of intrathoracic pressure.

Furthermore, in our patient laboratory signs of infection (elevated C-reactive protein) and thrombosis of the internal jugular vein was detected. On the one hand vein thrombosis is not always associated with catheter occlusion and can be asymptomatic [4]. On the other hand a removal of the catheter in case of deep vein thrombosis is recommended. To prevent thrombosis which is often associated with malignant disease, *Bern* et al. proposed a prophylactic treatment with 1 mg daily of warfarin in such patients [9]. The management of catheter-infection has been controversially discussed during the last decade. Initially, patients were treated with appropriate antibiotics and removal of the catheter, However, it is reported in the literature that patients can safely be treated with appropriate antibiotics alone without catheter removal [4,10]. In any case, the central venous catheter must be removed due to haemodynamic alterations of the patient or if a specific antibiotic therapy is not effective [11,12]. To prevent further complications an early explantation of a central venous access catheter should be discussed.

In conclusion because catheter migration might be asymptomatic, regular monitoring of the catheter position should be recommended. By any means prior to a new course of chemotherapy or encountering symptoms of migration, obtaining chest radiography is essential to provide early detection and to avoid consecutive complications. To prevent further complications an early explantation of a central venous access catheter should be suggested.

## List of abbreviations

CT: Computed tomography
ECF: pirubicin, cisplatin, 5-fluorouracil
I.U.: International unit
SVC: Superior vena cava
5-FU: 5-fluorouracil
